# Editorial Notes

**Published:** 1883-10

**Authors:** 


### EDITORIAL NOTES.

We are in receipt of a kind invitation to be present at the celebration of the one hundreth anniversary of the Medical School of Harvard University and the dedication of its new building.

The *Medico-Legal Journal* is one of the new ventures in journalism. It is the organ of the Medico-Legal Society of New York City, and promises to be a valuable addition to the list of American journals. The society is made up of distinguished members of the medical and legal profession, and has made many valuable contributions to jurisprudence and toxicology.

Among the numerous vehicles for the administration of quinnine in a tasteless form, syrup of licorice is by far the most effective, and the preparation of Gregory's, of "Syrup of Dover's" fame, seems to be particularly well fitted to accomplish the purpose. Three grains of quinine added to a teaspoonful of the syrup of licorice, Gregory's, makes a palatable mixture, the bitterness of the quinine being effectively masked.

We would call the attention of our readers to the advertisement of the *Duroy Native Wines.* We have given these wines careful trial and examination, and are convinced that the only addition to the pure juice of the grape is a little pure sugar, doubtless added to improve the keeping quality. We are glad to see that our principal druggists have these excellent wines in stock, as we believe them worthy of the entire confidence of the profession.

A good electrical battery is now a necessity to every physician. Among the many varieties offered, it is not easy for the uniniated to choose the best. From personal knowledge, we do not hesitate to commend the instruments furnished by the McIntosh & Faradic Galvanic Battery Co. Their combined galvanic and faradic battery is, take it all in all, the best and most convenient in all respects for the use of the physician now in the market.

